# Engineering Biomimetic Nanoparticle Performance Through Fabrication Method Selection: Turbulent Jet Mixing, Microfluidics, and Extrusion

**DOI:** 10.1002/smtd.202501770

**Published:** 2026-01-04

**Authors:** Ilana Elizarov, Rawan Mhajne, Ofri Vizenblit, Assaf Zinger

**Affiliations:** ^1^ Bioinspired Nano Engineering and Translational Therapeutics Lab Department of Chemical Engineering Technion−Israel Institute of Technology Haifa Israel; ^2^ Russell‐Berrie Nanotechnology Institute Technion – Israel Institute of Technology Haifa Israel; ^3^ Resnick Sustainability Center of Catalysis Technion−Israel Institute of Technology Haifa Israel; ^4^ Bruce and Ruth Rappaport Cancer Research Center Technion−Israel Institute of Technology Haifa Israel; ^5^ Cardiovascular Sciences Department Houston Methodist Academic Institute Houston TX USA; ^6^ Neurosurgery Department Houston Methodist Academic Institute Houston TX USA

**Keywords:** bioengineering, biomimicry, extrusion, microfluidics, nanoparticles design, turbulent jet mixing

## Abstract

Nanoparticle (NP) fabrication has advanced rapidly, driven by the growing role of nanomedicine in targeted drug delivery. Each fabrication strategy offers unique advantages and limitations. Here, we conduct a comparative evaluation of three prominent methods: turbulent jet mixing, microfluidic mixing, and extrusion, for producing biomimetic nanoparticles (BNPs). BNPs are emerging as next‐generation drug delivery platforms, combining liposomal biocompatibility with enhanced cellular uptake, prolonged circulation, and selective targeting, achieved by incorporating membrane proteins from source cells into synthetic lipid bilayers to confer cell‐mimicking functionality. Using neuron‐derived BNPs (“Neurosomes”) as a model, we systematically assess physicochemical and biological properties across fabrication methods and their impact on BNP function. Turbulent jet and microfluidic mixing produce BNPs with superior stability, higher membrane protein incorporation, improved batch‐to‐batch reproducibility, and enhanced targeting, whereas extrusion leads to diminished performance due to shear‐induced protein loss. Notably, this study presents the first application of high‐resolution LC‐MS/MS proteomics to quantitatively compare membrane‐associated protein profiles across fabrication methods. These results highlight the critical influence of fabrication techniques on BNP structure and function and provide actionable insights for optimizing production strategies, facilitating the scalable development of targeted nanotherapeutics.

## Graphical abstract

Biomimetic nanoparticles (BNPs) combine liposomal biocompatibility with cell‐mimicking functions for targeted delivery. This study compares turbulent jet mixing, microfluidic mixing, and extrusion for BNP fabrication. Characterization, proteomics, and in vitro assays show that turbulent jet and microfluidic mixing produce stable, protein‐enriched particles with enhanced functionality, while extrusion induces protein loss. These findings establish optimal strategies for scalable nanotherapeutic design.

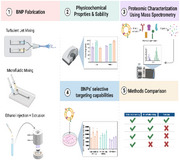

## 1 Introduction

Nanoparticle (NP) based platforms have become a cornerstone of drug delivery research, offering versatile strategies for therapeutic transport [1, 2]. Over the years, diverse fabrication techniques have been developed and optimized for various NP systems, including polymeric, gold, and liposomal NP [3]. Among these, liposomes have emerged as one of the most clinically advanced NP types, owing to their intrinsic biocompatibility, drug encapsulation capacity, and translational potential [4]. Despite their clinical success, conventional liposomes face persistent challenges such as rapid clearance by the mononuclear phagocyte system, limited tissue specificity, and suboptimal accumulation at target sites [5, 6]. To address these limitations, a new type of delivery system known as biomimetic nanoparticles (BNPs) has been developed [7]. BNPs are designed to mimic the surface characteristics of natural cells by incorporating biological components such as membrane proteins or entire cell membranes into synthetic lipid bilayers [8]. These bioinspired nano‐systems overcome complex physiological barriers and offering enhanced targeting specificity, prolonged systemic circulation, and improved immune evasion [1, 2, 8, 9, 7, 10, 11, 12, 13, 14].

Early BNP work demonstrated the feasibility of integrating biological components into synthetic vesicles, such as incorporating antigens from sheep erythrocyte membranes or bacterial lipopolysaccharides to create immune‐responsive nanoparticles for vaccine and immunomodulatory applications [15, 16, 17]. Following the clinical introduction of the first FDA‐approved liposomal drug, Doxil, in the late 1990s [18], the field shifted toward developing biomimetic, cell‐inspired delivery systems as therapeutic platforms. Since then, BNPs engineered from immune cells, cancer cells, stem cells, and neuronal cells have consistently demonstrated enhanced targeting specificity, homologous recognition, and improved cellular uptake compared to conventional liposomes [19, 20]. Across these studies, BNP performance has been tightly linked to physicochemical parameters such as size, polydispersity, membrane protein retention, and surface marker presentation, as well as biological metrics including uptake, biodistribution, and immune interactions.

BNPs can be broadly categorized into two main classes based on their membrane integration strategy. The first class involves the extraction of membrane proteins from specific cell types, which are subsequently reconstituted with synthetic lipid mixtures to form protein–lipid vesicles that emulate key cellular functions [8, 10, 21]. The second class employs whole plasma membranes isolated from donor cells to directly coat NP cores, such as PLGA [22], gold [23], or silica NP [24], resulting in membrane‐coated BNPs that preserve native membrane topology and surface markers. These strategies have generated promising BNP systems, yet systematic comparisons across fabrication methods remain limited, despite growing evidence that self‐assembly conditions influence BNP physicochemical properties, protein incorporation, and biological function.

BNP fabrication typically follows one of two approaches: **top‐down** or **bottom‐up**. In the top‐down approach, particles are first generated at the microscale and subsequently reduced to the nanoscale using mechanical methods such as extrusion or sonication [25]. This technique is most commonly used for fabricating membrane‐coated BNPs, starting with an intact cell membrane, coating the desired core [1]. In contrast, the bottom‐up approach assembles NP de novo by mixing purified membrane proteins with synthetic lipid mixtures. These components self‐assemble into vesicles through techniques such as microfluidic mixing [1]. This approach allows for precise control over parameters including aqueous‐to‐organic phase ratio, total flow rate, and lipid concentration, enabling tunable control over NP size, polydispersity, and protein incorporation efficiency. Moreover, bottom‐up fabrication offers high reproducibility, scalability, and design flexibility, making it well‐suited for programmable and modular BNPs platforms [13, 26].

In this study, we systematically compare fabrication strategies for generating BNPs composed of synthetic lipids and membrane proteins extracted from specific source cells (SH‐SY5Y neuroblastoma). Various self‐assembly techniques have been employed for BNPs fabrication, including extrusion, microfluidic mixing, and turbulent jet mixing. These methods differ significantly in their mixing dynamics, processing parameters, and scalability [26, 27, 28, 29, 30], all of which can influence the physicochemical properties, membrane protein incorporation, and biological performance of the resulting NPs. A comprehensive understanding of how fabrication methodology shapes BNPs composition and biological performance is therefore critical for the rational design and translation of these advanced drug delivery platforms.

One of the top‐down, traditional and basic methods for liposome production is **extrusion**. This method is typically used in conjunction with ethanol injection or thin‐film hydration to improve particle size uniformity. In the standard ethanol injection approach, first introduced by Batzri and Korn in 1973, lipids are dissolved in an organic solvent such as ethanol and rapidly injected into an aqueous solution. As ethanol disperses into water, the decreasing solubility of lipids drives the self‐assembly of vesicles. This process yields a heterogeneous mixture of small unilamellar vesicles (SUVs) and multilamellar vesicles (MLVs)[31]. To achieve uniform particle size and a narrower distribution, the suspension is subsequently extruded through polycarbonate membranes with defined pore sizes, yielding liposomes with controlled dimensions [32, 33]. While robust and straightforward, bench‐scale extrusion is labor‐intensive and inefficient, typically requiring ∼30 min for a 2 mL formulation and resulting in a 30%–50% sample volume loss [34]. Additionally, the method is poorly suited for delicate biological materials, as the high pressure and shear forces involved can damage membrane proteins and compromise BNPs integrity [35].

A widely adopted bottom‐up technique for BNPs and liposomal fabrication is **microfluidic mixing**, first introduced by Jahn et al. in 2010 [36]. This approach involves the simultaneous and precisely controlled injection of an organic phase (containing dissolved lipids) and an aqueous phase (containing membrane proteins, in the case of BNPs) into a microfluidic chip [10, 27]. Within the chip's microscale channels, the flow regime remains laminar, allowing for rapid and consistent mixing via diffusion. As the lipid solvent becomes diluted in the aqueous phase, spontaneous self‐assembly of lipid vesicles is triggered [37]. Key fabrication parameters‐ such as the flow rate ratio (FRR), total flow rate (TFR), and temperature‐ can be finely tuned to modulate particle size, polydispersity, and encapsulation efficiency. The resulting NP typically exhibit a narrow size distribution and high batch‐to‐batch reproducibility [10, 26, 38]. This technique is commonly implemented using platforms such as the NanoAssemblr Ignite, which utilizes disposable microfluidic cartridges and enables scalable, controlled, and reproducible NP fabrication suitable for both research and clinical development. Within the chip's microscale channels, the flow regime remains laminar, allowing for rapid and consistent mixing via diffusion. As the lipid solvent becomes diluted in the aqueous phase, spontaneous self‐assembly of lipid vesicles is triggered [1, 39].

Recently, **turbulent jet mixing** has emerged as a promising bottom‐up approach for NP fabrication and is being used more frequently [40, 41]. This method involves the rapid co‐injection of an organic phase (containing dissolved lipids) and an aqueous phase under high Reynolds number conditions (100 < Re < 2000), creating a confined turbulent mixing zone. The generated jet induces chaotic, multidirectional fluid motion, substantially enhancing mass transport and interfacial mixing between the two phases. This environment facilitates a dramatic increase in interfacial area, promoting the spontaneous self‐assembly of lipid‐based NP [30, 40, 41]. Turbulent jet mixing is implemented using specially designed mixers or modified microfluidic devices. Key parameters such as flow rate (which determines the Reynolds number), solution composition, and device geometry influence the resulting NP size and uniformity [30]. The method is particularly attractive due to its simplicity, scalability, and efficiency in producing NP with desirable physicochemical properties [30, 40, 41]. Notably, unlike traditional microfluidic systems that rely on disposable chips, turbulent jet mixing employs a reusable jet mixer, enabling cost‐effective, high‐throughput production without compromising formulation quality. This reusability offers a significant advantage for translational and industrial‐scale manufacturing of nanomedicines.

Here, we present a systematic comparison of three self‐assembly techniques‐ turbulent jet mixing, microfluidic mixing, and extrusion‐ for the fabrication of BNPs composed of a defined lipid bilayer and membrane proteins extracted from SH‐SY5Y neuroblastoma cells. These neuron‐derived BNPs, termed Neurosomes, were previously developed by Zinger et al. [8] and shown to exhibit enhanced targeting and uptake by neural cells relative to conventional liposomes, owing to their improved cell‐specific recognition and membrane mimicry [8].

In this study, Neurosomes were selected as a representative example of biomimetic nanoparticles platform to evaluate how fabrication technique impacts structural and functional performance. We evaluated the resulting BNPs in terms of their physicochemical properties under both storage conditions and physiological conditions, evaluating their stability, and determining doxorubicin encapsulation efficiency and release behavior. Membrane protein incorporation and batch‐to‐batch reproducibility were quantified using high‐resolution, label‐free LC–MS/MS proteomic analysis, enabling comparison of membrane‐associated protein retention across fabrication strategies. Finally, biomimetic functionality was examined through an in vitro homotypic association assay with SH‐SY5Y neuronal cells to determine how fabrication affects cell–specific interactions.

This study provides the first direct comparison of protein–lipid BNPs fabrication strategies and offers mechanistic insights into how different self‐assembly techniques affect protein loading, structural integrity, and biomimetic functionality, guiding the rational design of next‐generation nanocarriers for targeted drug delivery.

## 2 Results

To identify the optimal fabrication method for BNPs, neuron‐derived BNPs (Neurosomes), were produced using three techniques: turbulent jet mixing, microfluidic mixing, and extrusion. In all cases, BNPs were formulated using membrane proteins extracted from SH‐SY5Y neuroblastoma cells, with a fixed protein‐to‐lipid mass ratio of 1:50. As controls, empty liposomes were prepared using the same fabrication methods but without protein incorporation. The selected protein: lipid (P:L) ratio was based on experimentally defined physicochemical constraints established prior to fabrication [8, 10]. In this platform, acceptable formulations required size of < 200 nm, PDI < 0.2, maintenance of a negative zeta potential after protein incorporation, preservation of bilayer integrity, and retention of neuronal surface markers. In previous optimization studies, the protein suspension buffer was found to strongly influence particle size and PDI, such that the highest P:L ratio that maintained all required criteria corresponded to a practical upper limit of 1:20. Ratios above this threshold disrupted nanoparticle homogeneity and bilayer stability. A ratio of 1:50 therefore falls well within the validated stability‐permissive window, ensuring robust reproducibility while supporting effective protein incorporation. Although higher protein loading can potentially enhance biomimetic function, such ratios are not physically achievable in this system due to buffer‐dependent destabilization at P:L values above 1:20. The 1:50 ratio was thus selected as a scientifically justified balance between stability, uniformity, and biological relevance across all fabrication methods.

Optimal fabrication parameters for the Microfluidic and Extrusion methods were selected based on previously reported protocols [8, 10, 13]. For the Turbulent Jet mixing method, calibration experiments were performed using liposome formulations at total flow rates (TFR) of 2.5, 3, and 4 mL/min. As shown in Figure S1, increasing the flow rate led to a reduction in particle size. A TFR of 3 mL/min was selected as it produced nanoparticles with the desired size of approximately 100 nm.

All NP formulations were systematically characterized with respect to their physicochemical properties, lipid bilayer integrity, stability in physiological conditions, drug loading efficacy, protein content and proteomic profile, and in vitro targeting capability‐key indicators of their biomimetic functionality.

### 2.1 Physiochemical Characterization of BNPs Formulations

The physiochemical properties of the NP formulations were evaluated using dynamic light scattering (DLS) to measure hydrodynamic diameter, polydispersity index (PDI), particle concentration, and zeta potential. Cryogenic transmission electron microscopy (Cryo‐TEM) was employed to assess particle morphology and verify the presence of a well‐defined lipid bilayer. In parallel, formulation stability was monitored over 21 days at 4°C.

**FIGURE 1:**
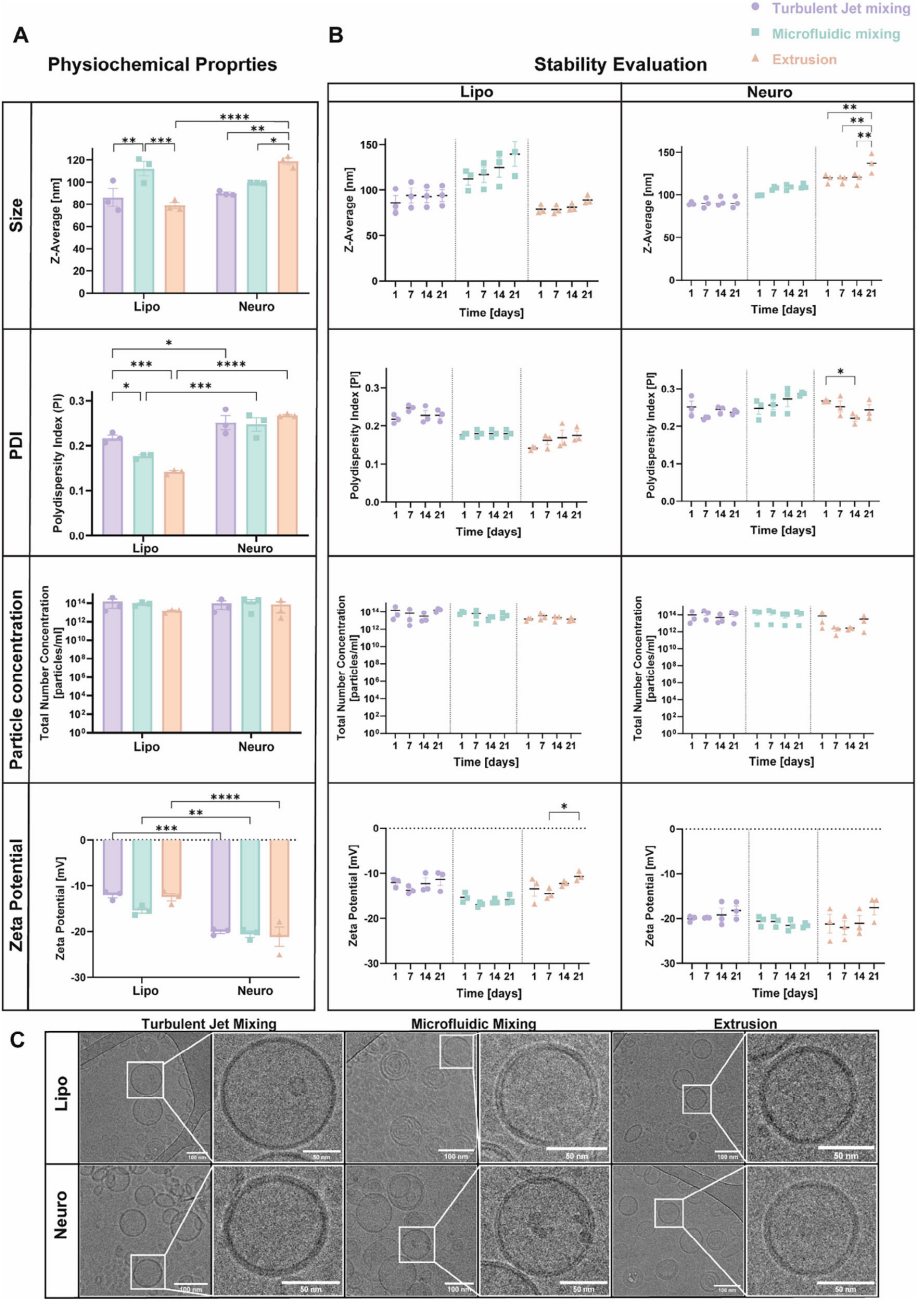
Physiochemical Characterization and Stability of Neurosomes Formulations. (A) Dynamic light scattering (DLS) analysis of Liposomes and Neurosomes fabricated via turbulent jet mixing, microfluidic mixing, or extrusion. Key physicochemical parameters assessed include particle size, polydispersity index (PDI), particle concentration, and zeta potential. (B) Stability of Neurosome formulations over 21 days at 4°C, monitored by DLS. (C) Representative cryo‐TEM images confirming spherical morphology and lipid bilayer integrity across all fabrication methods. Scale bar: 50 nm & 100 nm. Data are presented as mean ± SEM (n = 3 per formulation). Statistical analysis was performed using two‐way ANOVA followed by Tukey's multiple comparison test: ^*^ *p* < 0.05, ^**^ *p* < 0.01, ^***^ *p* < 0.001, ^****^ *p* < 0.0001.

As shown in Figure 1A, liposomes exhibited particle sizes ranging from 85–115 nm, while Neurosomes ranged from 90–120 nm. No significant size differences were observed between liposomes and Neurosomes fabricated via microfluidic or turbulent jet mixing. However, extrusion‐based fabrication yielded markedly inconsistent sizes: liposomes averaged 75 nm, whereas Neurosomes reached approximately 120 nm. This discrepancy underscores the limitations of extrusion, which produced variable particle sizes when membrane proteins were incorporated, suggesting incompatibility between this method and BNPs formulation under identical conditions. Additional optimization would be required to achieve consistent size distributions across both protein‐loaded and empty NP formulations.

PDI values, which indicate the uniformity of particle size distribution, remained below 0.3 across all formulations, indicating relatively uniform particles populations. However, within each fabrication method, Neurosomes consistently exhibited higher PDI values compared to their liposomal counterparts. This expected increase in heterogeneity is attributed to membrane protein incorporation, which can disrupt the self‐assembly process. Among the liposomal formulations, extrusion yielded the lowest PDI values, consistent with prior reports demonstrating its effectiveness in producing homogeneous conventional liposomes [30]. In contrast, this benefit did not extend to Neurosomes, further demonstrating extrusion's limitations for protein‐containing BNPs.

Microfluidic mixing produces liposomes with lower PDI values than turbulent jet mixing, suggesting greater formulation uniformity. This may be due to more precise thermal regulation since microfluidic systems allow tight control of temperature near the lipid transition temperature (Tm), optimizing lipid fluidity during self‐assembly. In contrast, turbulent jet mixing in this study relied on an external water bath without integrated temperature control, which may have contributed to broader size distributions. Nevertheless, turbulent jet mixing still produced NP with PDI values well within the acceptable threshold (< 0.3), supporting its feasibility for research and potential clinical applications.

Particle concentrations ranged from 1.5 × 10^13^ to 1.5 × 10^14^ particles/mL across all fabrication methods, indicating comparably high yields regardless of technique. Zeta potential measurements revealed more negative surface charges for Neurosomes (−18–−21 mV) compared to liposomes (−11–−15 mV), likely reflecting the presence of membrane proteins that increase surface charge density, as previously demonstrated by Zinger et al. [7, 8, 10].

Stability data, presented in Figure 1B, indicate that BNPs fabricated by microfluidic and turbulent jet mixing remained stable over the 21‐day storage period at 4°C, with minimal changes in particle size, PDI, concentration, or zeta potential. In contrast, Neurosomes produced by extrusion exhibited significant fluctuations in all parameters, indicating poor colloidal stability.

Cryo‐TEM images (Figure 1C) provide macro view of NPs populations and confirm the presence of spherical, uniformly shaped NP across all formulations and fabrication methods. Importantly, a well‐defined lipid bilayer structure was preserved in both liposomes and Neurosomes, validating the integrity of the vesicular architecture.

### 2.2 Assessment of BNP Physiological Stability and Doxorubicin Encapsulation Efficiency and Release Profile

The potential of BNPs as a drug delivery platform was evaluated through assessment of their stability under physiological conditions and their capacity for drug loading. As a proof of concept, doxorubicin (Dox) was encapsulated within both liposomes and Neurosomes prepared using different fabrication methods. Dox was selected as a chemotherapeutic model to demonstrate the feasibility of BNP‐mediated delivery for brain cancer therapy, particularly glioblastoma (GBM) [42, 43]. The therapeutic use of free Dox is limited by its poor permeability across the blood–brain barrier (BBB) [44, 45], despite demonstrated efficacy in preclinical and clinical studies [42, 46, 47]. Since Neurosomes are designed to cross the BBB, their use for Dox delivery may offer a promising strategy for targeted and effective treatment of neurological diseases.

To examine the physicochemical robustness and loading performance of these systems, two complementary analyses were conducted. First, the stability of empty nanoparticles fabricated by different methods was evaluated under physiological conditions (SH‐SY5Y growth medium containing DMEM and 10% serum) at 37°C for 0 (baseline), 6, 24, and 48 h. A maximum incubation duration of 48 h was selected because liposomal particles are typically cleared from the bloodstream within this timeframe in vivo [48, 49]. Second, to assess the encapsulation capability of the BNPs, particles were loaded with Dox using an active loading approach, and the Dox release profile was subsequently measured at 4°C and 37°C over 8, 24, 48, and 72 h.

**FIGURE 2:**
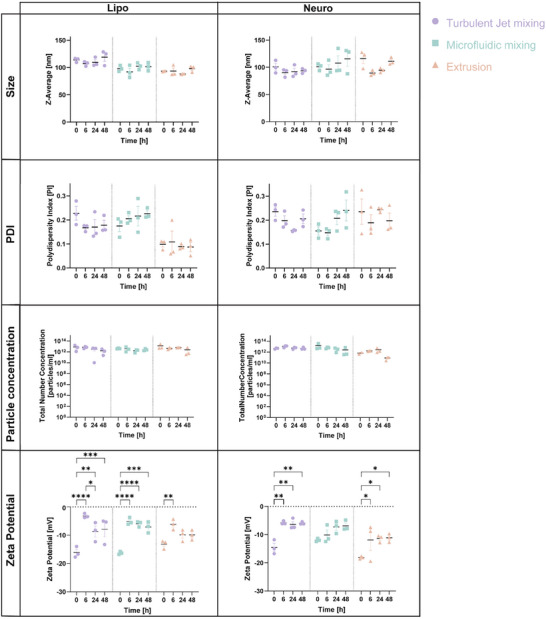
BNP stability under physiological conditions. Dynamic light scattering (DLS) analysis of empty liposomes and Neurosomes fabricated via turbulent jet mixing, microfluidic mixing, or extrusion. BNPs were incubated in SH‐SY5Y growth medium at 37°C for 0, 6, 24, and 48 h to assess their stability under physiological conditions. Key physicochemical parameters measured included particle size, polydispersity index (PDI), particle concentration, and zeta potential. Data are presented as mean ± SEM (n = 3 per formulation). Statistical analysis was different fabrication methods using two‐way ANOVA followed by Tukey's multiple comparisons test: ^*^ *p* < 0.05, ^**^ *p* < 0.01, ^***^ *p* < 0.001, ^****^ *p* < 0.0001.

Results in Figure 2 demonstrated that for both Liposome and Neurosome formulations, size, PDI, and particle concentration remained relatively stable over 48 h for all fabrication methods, indicating robust colloidal stability in biological media. However, statistically significant differences in zeta potential were detected among the fabrication methods at several time points. A decrease in the absolute zeta potential was observed, reflecting reduced colloidal stability and increased particle aggregation. This reduction is attributed to the adsorption of serum proteins onto the nanoparticle surface, which forms a protein corona and partially masks the native negative surface charge [50, 51, 52].

**FIGURE 3:**
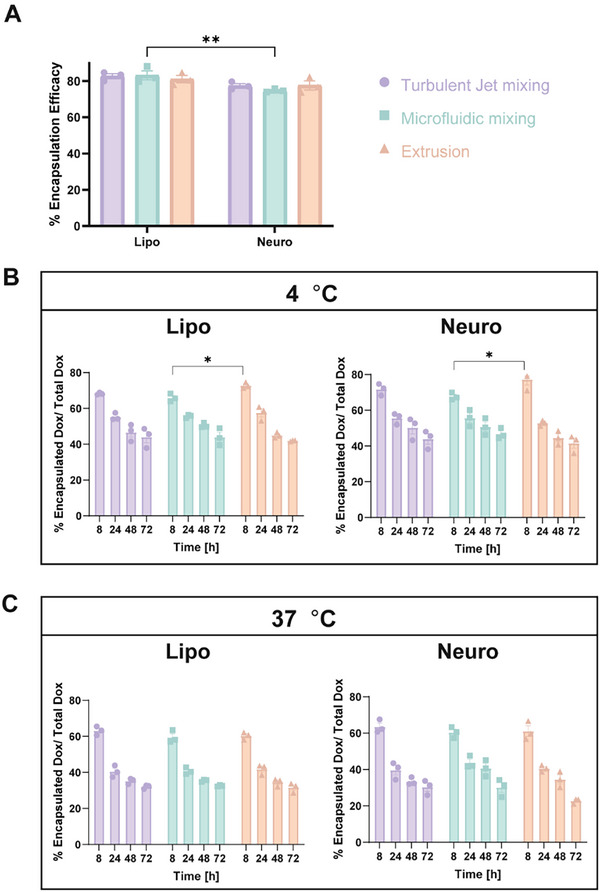
Doxorubicin Encapsulation Efficiency and Release Profile of BNPs. (A)Doxorubicin encapsulation efficiency of each formulation and cumulative release profiles measured at 4°C (B) and 37°C (C) over 8, 24, 48, and 72 h. Data are presented as mean ± SEM (n = 3 per formulation). Statistical significance is shown only for comparisons between groups and was evaluated using a two‐way ANOVA followed by Tukey's multiple comparisons test. Significance levels are indicated as follows: ^*^ *p* < 0.05; ^**^ *p* < 0.01; ^***^ *p* < 0.001; ^****^ *p* < 0.0001. Full statistical analysis, including within‐group comparisons, is provided in Figure S3.

Drug encapsulation analysis (Figure 3A) showed that all formulations achieved approximately 80% Dox loading, indicating efficient encapsulation across fabrication methods. At 4°C (Figure 3B; Figure S3A), both liposome and Neurosome formulations retained most of the encapsulated Dox over 72 h, although statistically significant decrease in retention was observed. At 37°C (Figure 3C; Figure S3B), Dox retention declined significantly more rapidly for all BNP types and fabrication approaches, consistent with enhanced drug release under physiological conditions.

Overall, these results indicate that both liposome and Neurosome possess robust physicochemical stability and effective drug‐loading capabilities, supporting their feasibility as carriers for controlled Dox delivery and highlighting their potential for therapeutic applications in the central nervous system.

### 2.3 Evaluation of Protein Content and Reproducibility in BNPs Fabricated by Different Methods

Following physicochemical and morphological characterization, the protein content and reproducibility of BNPs were evaluated, with a particular emphasis on membrane protein incorporation and consistency across replicates for each fabrication method.

**FIGURE 4:**
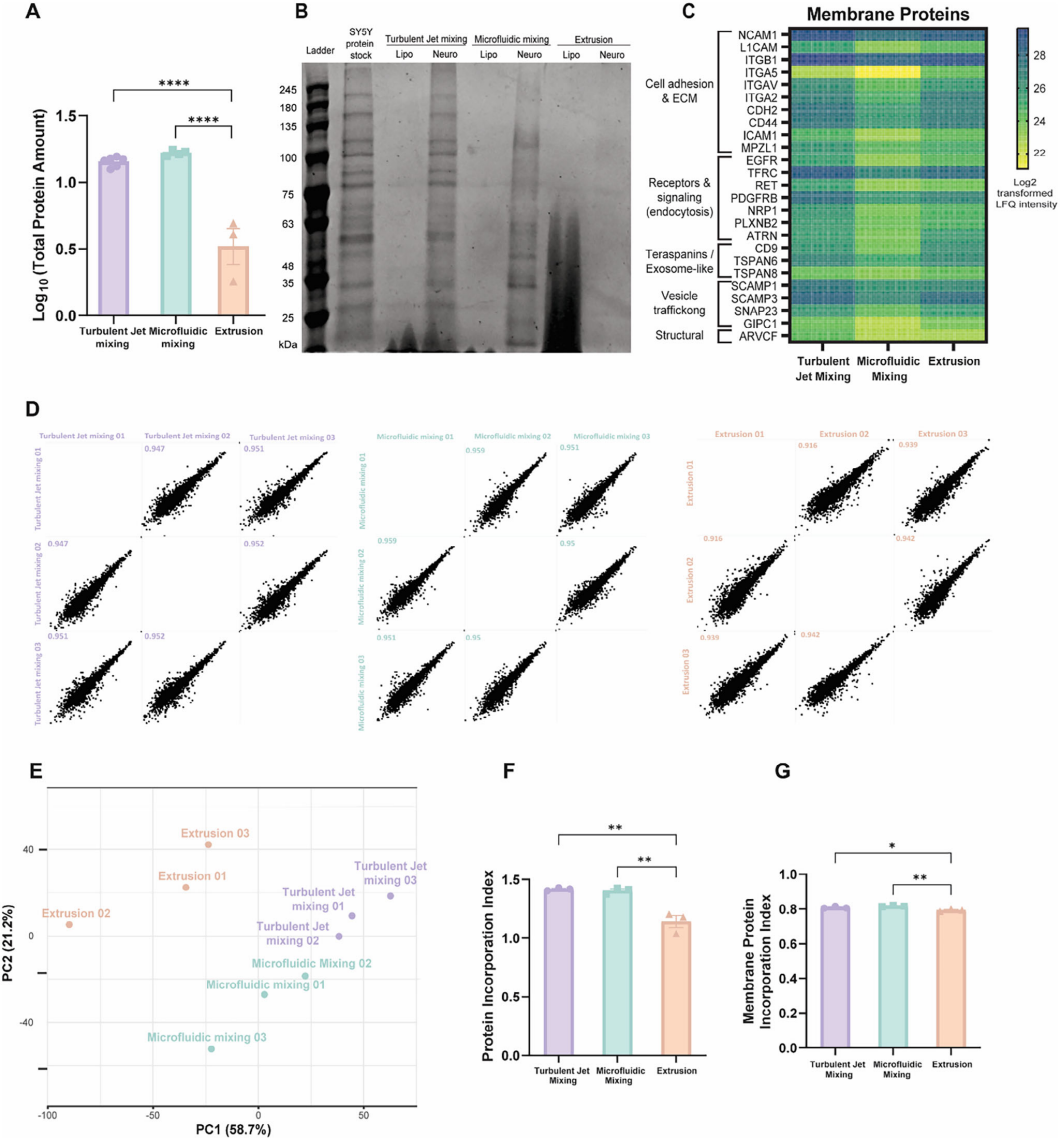
Protein Content and Reproducibility of BNPs Fabricated by Different Methods. (A) Log_10_‐transformed total protein amount extracted from 200 µL of nanoparticles prepared by each method (µg), as quantified by Bradford assay. The raw data is available in Table S1. Bars represent mean ± SEM (n = 3). Statistical analysis was performed using one‐way ANOVA with Tukey's multiple comparison test: ^****^ *p* < 0.0001. (B) SDS‐PAGE analysis comparing the protein stock used for BNPs fabrication, plain liposomes, and Neurosomes generated by each method. Equal NP volumes were loaded per lane. Distinct protein bands are visible for turbulent jet and microfluidic samples, whereas extrusion samples resemble plain liposomes, indicating reduced protein incorporation. (C) Heatmap showing the relative abundance of representative membrane proteins incorporated into Neurosomes. Protein abundance is represented as the average of transformed LFQ intensity. (D) Multi‐scatter plots of protein intensity values from three independent replicates of Neurosomes fabricated via each method. Pearson correlation coefficients (R) indicate high intra‐group reproducibility for turbulent jet and microfluidic mixing (R ≥ 0.95), and slightly lower reproducibility for extrusion (R  =  0.90–0.95). (E) Principal component analysis (PCA) of protein composition in Neurosomes fabricated by each method. PC1 and PC2 account for 58% and 21% of total variance, respectively. Replicates from turbulent jet and microfluidic mixing clustered tightly, indicating consistent protein composition. (F) Identity‐based incorporation analysis. The percentage of proteins in each BNPs formulation that overlapped with the original input stock was calculated using label‐free quantification intensity (LFQ‐intensity). To meet ANOVA assumptions, values were first converted to proportions (0–1) and then arcsine square root‐transformed before statistical analysis. The raw data is provided in Table S2. Statistical significance determined via one‐way ANOVA with Tukey's post hoc test: ^**^ *p* < 0.01. (G) Relative abundance of membrane‐associated proteins in BNPs formulations. The total signal intensity of membrane proteins was divided by the total protein signal to estimate membrane protein proportion. The original protein stock (not shown) contained ∼52% of membrane proteins and served as a reference. To meet ANOVA assumptions, values were first converted to proportions (0–1) and then arcsine square root‐transformed before statistical analysis. The raw data is provided in Table S3. Statistical significance determined via one‐way ANOVA with Tukey's post hoc test: ^*^ *p* < 0.05, ^**^ *p* < 0.01. Proteomic data processing details are provided in the Methods section.

Total protein content was first quantified following extraction by acetone precipitation. BNPs generated via turbulent jet mixing and microfluidic mixing incorporated significantly greater amounts of protein, approximately 14–16 µg per formulation, compared to those produced via extrusion, which incorporated only ∼3.5 µg (Figure 4A; Table S1). These findings indicate that protein incorporation efficiency is substantially lower using extrusion method compared to the other two methods.

SDS‐PAGE analysis (Figure 4B) further supports these findings. Equal NP volumes from each group were loaded onto the gel. BNPs produced via turbulent jet and microfluidic mixing exhibit distinct protein bands, with intensity and migration patterns similar to those observed in the original protein stock used for fabrication. In contrast, extrusion‐based BNPs showed no visible bands, similar to plain liposomes, suggesting either poor incorporation or possible protein degradation during the extrusion process. This observation is reinforced by Figure S2, which reveals a visible pink smear on the polycarbonate membrane following extrusion of Neurosomes‐likely indicative of protein loss during processing. Mechanistically, the improved protein retention is attributed to the gentle self‐assembly dynamics of microfluidic mixing and enhanced interfacial mixing in turbulent jet systems, whereas the high shear stress in extrusion may lead to protein denaturation or shedding.

Heat maps of representative membrane‐associated and non‐membrane proteins from SH‐SY5Y cells are shown in Figure 4C and Figure S4, illustrating the protein identities incorporated on nanoparticles. The membrane protein map (Figure 4C) highlights proteins involved in cell adhesion, neuronal communication, endocytosis, trafficking, and neuronal targeting. Classical adhesion and extracellular matrix (ECM) binding proteins, including *NCAM1, L1CAM, ITGB1, ITGA5, ITGAV, ITGA2, CDH2, CD44, ICAM1*, and *MPZL1*, contribute to membrane interactions and ECM binding, emphasizing their potential to mediate liposome attachment via extracellular interactions [53, 54]. Receptors and signaling proteins such as *EGFR, TFRC, RET, PDGFRB, NRP1, PLXNB2*, and *ATRN* facilitate receptor‐mediated internalization, supporting the liposomes’ biological effects on target cells [54]. Tetraspanins and trafficking regulators (*CD9, TSPAN6, TSPAN8*) are critical for membrane organization, trafficking, and targeting, while additional trafficking proteins (*SCAMP1, SCAMP3, SNAP23, GIPC1*) and junctional proteins (*ARVCF*) contribute to structural regulation of vesicular transport [55]. Importantly, previous proteomic and flow cytometric studies have confirmed surface expression of all these proteins on SH‐SY5Y neuroblastoma cells, underscoring their relevance to the neuroblastoma phenotype and their potential as functional proteins for targeted drug delivery applications [54, 56]. These patterns provide a mechanistic basis for targeted delivery of liposomes incorporating neuronal membrane proteins to SH‐SY5Y cells.

In contrast, the non‐membrane protein map (Figure S4) includes representative proteins that commonly co‐purify with membrane and considered minor contaminants, reflecting the inherent limitations in the purification efficiency of the protein extraction process [57]. This group consists mainly of ribosomal, mitochondrial, and nuclear proteins that commonly co‐purify with membrane fractions due to their high cellular abundance, partial association with intracellular membranes, or general stickiness during extraction [58, 59, 60, 61]. Ribosomal proteins often appear because ribosomes are physically linked to the endoplasmic reticulum, mitochondrial proteins tend to enrich in lipid‐rich fractions; and nuclear proteins typically result from nuclear leakage during lysis and can precipitate with membrane material. Additional cytosolic proteins, such as structural, stress‐response, and metabolic enzymes, represent expected background contaminants originating from the overall cellular protein pool. Together, these proteins are interpreted as co‐purifying species rather than true plasma‐membrane components.

Overall, these proteomic data presented to identify the proteins incorporated into each BNP formulation and to better understand the resulting protein profiles, thereby providing insights into their potential contribution to the biological mechanism of targeted delivery.

To investigate protein composition and membrane protein retention more comprehensively, label‐free quantitative proteomic analysis (LC‐MS/MS) was performed. Equal total protein amounts from three independent replicates per fabrication method were analyzed. Protein composition reproducibility was assessed by comparing relative signal intensities across replicates using multiscatter plots and Pearson correlation coefficients. BNPs fabricated by turbulent jet and microfluidic mixing exhibited high reproducibility, with correlation coefficients exceeding 0.95. In contrast, extrusion‐based BNPs displayed lower reproducibility, with coefficients ranging from 0.90 to 0.95 (Figure 4D).

These findings were further validated through principal component analysis (PCA) (Figure 4E). Replicates from turbulent jet and microfluidic mixing clustered tightly, indicating similar protein compositions. In contrast, extrusion replicates were more dispersed, reflecting increased variability in protein incorporation.

To evaluate the efficiency of protein incorporation from the original stock into the BNPs, the shared proteins between the stock and each BNP formulation were analyzed using label‐free quantification (LFQ) intensities. LFQ is a relative quantification approach in proteomics that enables comparison of protein abundance across different samples by normalizing peptide intensities and accounting for technical variation [62]. This method provides insight into how consistently proteins from the input stock are incorporated into the nanoparticles across replicates (Figure 4F). Over 97% of the proteins present in the input stock were retained in BNPs fabricated via turbulent jet and microfluidic mixing, whereas extrusion‐based BNPs retained only ∼82%, with higher variability among replicates; consistent with the trends seen in the multiscatter plots and PCA (Table S2).

To further examine the incorporation of membrane‐associated proteins‐key contributors to the cell‐mimicking properties of biomimetic nanoparticles (BNPs)‐we quantified their relative abundance using intensity‐based absolute quantification (iBAQ) values. iBAQ is a label‐free proteomics metric that estimates the relative molar abundance of proteins by dividing the summed peptide intensities by the number of theoretically observable peptides for each protein, thereby allowing comparison across different proteins within a sample [62]. In the input stock, membrane proteins accounted for ∼52% of the total signal. This proportion was consistently maintained across all BNPs formulations, ranging from 50% to 54% (Figure 4G; Table S3), indicating no significant enrichment or depletion of membrane proteins across fabrication methods. However, formulations generated via turbulent jet and microfluidic mixing exhibited slightly higher membrane protein retention than those produced by extrusion, suggesting modestly improved preservation of biologically relevant components.

### 2.4 Evaluation of Neurosome–Cell Association and Cytocompatibility in SH‐SY5Y Cell Culture

To evaluate the targeting potential of Neurosomes toward neuronal cells via homotypic cell‐to‐cell communication, BNPs fabricated using turbulent jet mixing, microfluidic mixing, and extrusion were incubated with SH‐SY5Y neuroblastoma cells for 4, 12, and 24 h at lipid concentrations of 0.1 and 0.5 mm. It is important to note that the orientation of membrane proteins on the BNP surface is crucial for functional targeting. Previous work by Zinger et al. [10] demonstrated that approximately 50% of membrane proteins are outward‐facing in self‐assembled BNPs. Since all fabrication methods in our study rely on analogous self‐assembly processes and similar formulations, we expect a comparable protein orientation in our Neurosome formulations, ensuring functional display of key membrane proteins. Nanoparticle cell association was quantified by measuring fluorescence intensity using a Cytation 5 microplate reader. In parallel, cytocompatibility was assessed using the MTT assay to determine the proportion of viable cells relative to untreated controls. To complement these experiments, previously published data from Zinger et al. demonstrated that Neurosomes do not exhibit increased association with non‐neuronal CNS cell types such as human astrocytes and microglia (Figure S4), supporting their preferential interaction with neuronal cells [8, 63].

**FIGURE 5:**
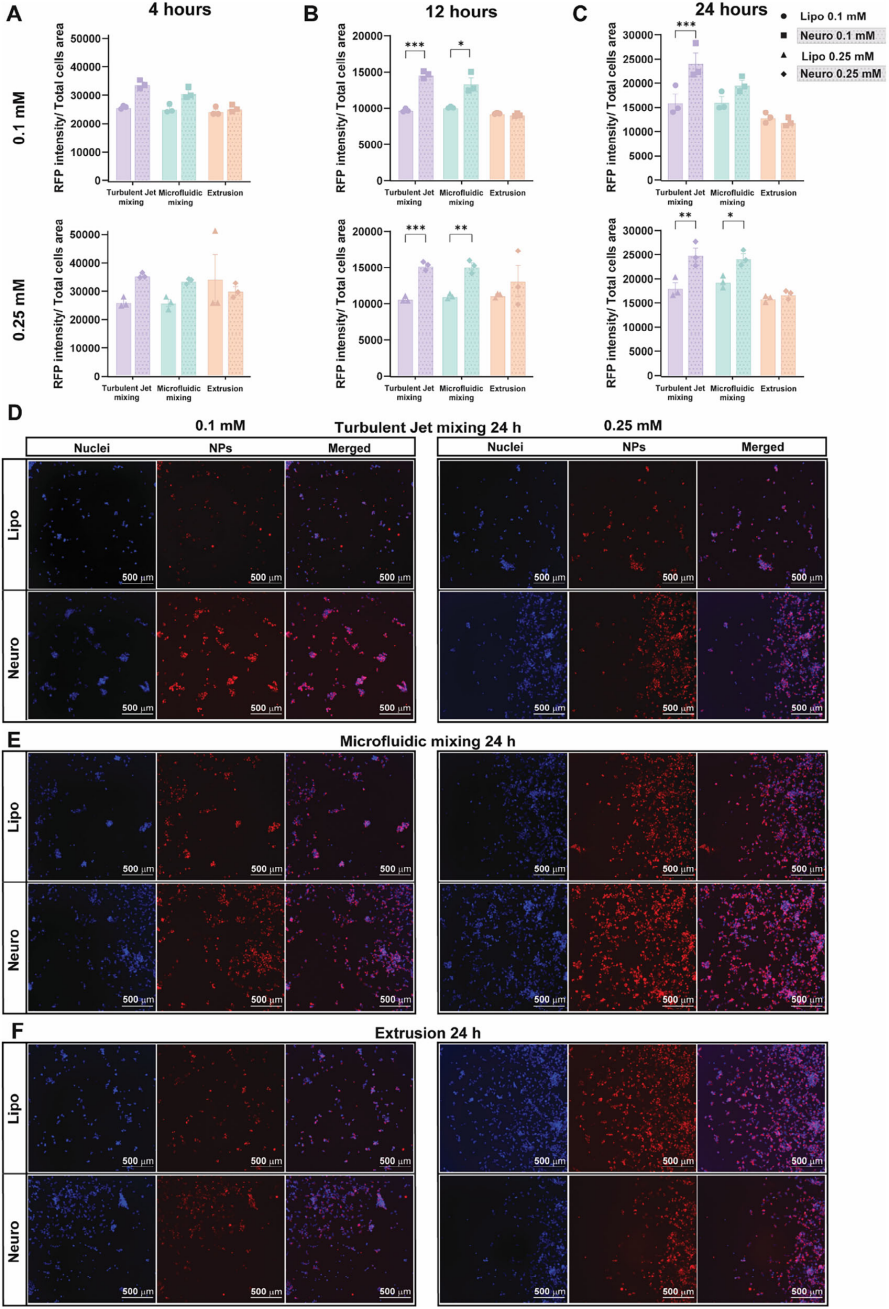
Association of Neurosomes with SH‐SY5Y Neuronal Cells. (A–C) Quantification of NP association with SH‐SY5Y neuroblastoma cells following incubation with Neurosomes for 4 h (A), 12 h (B), and 24 h (C), fabricated via turbulent jet mixing, microfluidic mixing, or extrusion. Fluorescence intensity was measured to assess NP–cell interaction at two lipid concentrations (0.1 and 0.5 mm). (D–F) Representative fluorescence microscopy images of SH‐SY5Y cells incubated for 24 h with rhodamine‐labeled Neurosomes (red) fabricated by turbulent jet mixing (D), microfluidic mixing (E), or extrusion (F). Cell nuclei were stained with DAPI (blue). Scale bar: 500 µm. Data are presented as mean ± SEM (n = 3 per formulation). Statistical analysis was performed using two‐way ANOVA with Tukey's multiple comparison test: ^*^ *p* < 0.05, ^**^ *p* < 0.01, ^***^ *p* < 0.001, ^****^ *p* < 0.0001.

After 4 h of incubation, no significant differences in cellular association were observed between Neurosomes and liposomes across all fabrication methods (Figure 5A), suggesting that short exposure times are insufficient to reveal the enhanced interaction conferred by biomimetic surface features. However, at 12 and 24 h, Neurosomes fabricated by microfluidic mixing and turbulent jet mixing exhibited significantly greater association with SH‐SY5Y cells compared to their liposome's counterparts at both concentrations (Figure 5B,C,D,E). These results indicate that, with sufficient incubation time, the membrane protein components of Neurosomes facilitate enhanced neuronal uptake via cell‐specific interactions.

In contrast, no significant difference in cell association was observed for Neurosomes and liposomes produced by the extrusion method, even at extended incubation times (Figure 5B,C,F).

Cytocompatibility data (Figure S5) demonstrated that all formulations, both liposomes and Neurosomes, were well tolerated by SH‐SY5Y cells. The obtained viability ratios (relative to non‐treated controls) indicate that most formulations maintained high cell viability, with extrusion‐based BNPs exhibiting a slightly lower compatibility that was not statistically significant.

### 2.5 Comparative Summary of Fabrication Methods for BNPs

Table 1 provides a comparative overview of the key characteristics associated with the three fabrication methods evaluated in this study: turbulent jet mixing, microfluidic mixing, and extrusion. The summary incorporates both our experimental findings and relevant data from previous literature, and includes additional considerations such as cost, ease of use, and adaptability to different proteins and lipid compositions. Key parameters considered include sample processing volume, waste generation, device reusability, material compatibility, processing time, temperature control, and protein incorporation efficiency. These criteria collectively influence the scalability, reproducibility, and functional output of BNPs formulations.

**TABLE 1:** Comparative summary of fabrication methods for BNPs.

	Turbulent mixing	Microfluidic	Extrusion
Sample volume per run	200 µL–100 mL [28]	1 mL–60 mL [39]	1 mL–10 mL [29]
Waste volumes (for 2 mL formulation)	450 µL	500 µL	750–1000 µL
Device reusability	Multiple‐use mixer	Single‐use microfluidic chip	Reusable extruder filter system
Material compatibility	Broad solvents	Limited solvents	Broad solvents
Production time (for 1 mL formulation)	∼1 min	∼1 min	∼10–45 min
Temperature control	Not available	Available	Available
Protein incorporation	Similar to the protein stock	Similar to the protein stock	Low protein loading due to shear forces and pressure
Cost	Moderate, specialized equipment	High, specialized equipment, single use chip	Low, standard lab equipment
Ease of use	Moderate, requires training	Moderate, requires chip handling and requires training	Moderate, straightforward and requires training
Adaptability to proteins/lipids	High, broad compatibility	Moderate, sensitive to solvent type	Limited, sensitive to protein integrity

Turbulent jet mixing offers rapid production, broad solvent compatibility, high protein retention, and reusable equipment, making it suitable for high‐throughput preclinical studies. However, it provides limited temperature control and requires specialized equipment, which may increase setup costs. Microfluidic mixing enables precise control over vesicle size and homogeneity, with excellent reproducibility and moderate protein retention. Its main limitations include higher costs due to single‐use chips (up to November 2025) and restricted solvent compatibility, although it is well‐suited for sensitive protein–lipid combinations. Extrusion is simple and low‐cost, compatible with a wide range of solvents, and effective for producing homogeneous conventional liposomes. However, it is labor‐intensive, time‐consuming, and exhibits lower protein incorporation due to shear stress, limiting its suitability for complex BNPs.

This comparison offers a practical reference for researchers seeking to select the most suitable fabrication approach for BNPs production, balancing performance, scalability, and translational potential.

## 3 Conclusion

This study presents a systematic, comparative evaluation of three fabrication strategies‐ turbulent jet mixing, microfluidic mixing, and extrusion‐ for producing neuron‐derived BNPs (“Neurosomes”) and assessing their effects on the functional capabilities of Neurosomes as representative BNPs. Through a comprehensive assessment of physicochemical properties, stability under storage and physiological conditions, doxorubicin encapsulation and release, protein incorporation efficiency, proteomic consistency, and in vitro functionality, we demonstrate that turbulent jet mixing and microfluidic mixing consistently outperform extrusion in multiple key performance metrics.

Neurosomes fabricated via turbulent jet and microfluidic mixing exhibited enhanced colloidal stability and reproducibility compared to those produced by extrusion. Stability studies under physiological conditions confirmed that these formulations maintain size, polydispersity, and protein content over time in biologically relevant media, whereas extrusion‐derived particles showed greater aggregation and variability. While extrusion yielded lower PDI values for simple liposomes, uniformity was compromised in protein‐containing BNPs, likely due to shear‐induced protein denaturation. Microfluidic mixing produced narrower size distributions, reflecting precise control over thermal and flow conditions, whereas turbulent jet mixing produced slightly higher PDI values but maintained reproducibility and protein content. Intra‐group variability observed across all methods highlights the importance of standardized protocols to minimize user‐dependent differences.

While the PDI values obtained remained below 0.3, it should be noted that they fall outside the ISO 22412:2025 recommended range of 0.05–0.20 for liposomal formulations intended for pharmaceutical production [64]. It should also be emphasized that this ISO standard is designed for final, clinical‐grade products, whereas biomimetic systems at the research stage, particularly those incorporating complex membrane protein mixtures, are commonly characterized by higher PDI values due to intrinsic membrane heterogeneity [65, 66, 67]. Despite exceeding the ISO threshold, the PDI values observed in this study are still within the ranges reported across published BNP formulations and are therefore considered appropriate for preclinical research, mechanistic investigations, and early translational development [68]. It is anticipated that further optimization, such as the implementation of more precise temperature control or modifications to mixer geometry, could reduce PDI values in future formulations to more closely align with clinical manufacturing specifications.

Doxorubicin encapsulation and sustained release were achieved across all fabrication methods, demonstrating that both bottom‐up and top‐down approaches support integration of therapeutic payloads while maintaining BNP structural integrity. Notably, turbulent jet and microfluidic formulations retained high encapsulation efficiency without compromising membrane protein functionality, illustrating the feasibility of concurrent drug delivery and biomimetic surface presentation.

Protein incorporation and proteomic analysis revealed that turbulent jet and microfluidic mixing achieved high total protein loading and preservation of membrane‐associated proteins, whereas extrusion yielded lower incorporation due to mechanical stress. Mechanistically, these findings suggest that reduced shear stress and controlled mixing dynamics facilitate retention of functional membrane proteins, which directly impacts homotypic targeting capability and cellular interactions.

Functionally, only Neurosomes produced via turbulent jet and microfluidic mixing demonstrated enhanced association with SH‐SY5Y neuronal cells, consistent with higher membrane protein content. These results highlight that fabrication method influences not only physicochemical and proteomic properties but also biologically relevant biomimetic functionality.

From a translational perspective, scalability and reproducibility differ markedly between methods. Microfluidic mixing offers highly reproducible, precisely controlled formulations but is constrained by single‐use chips and moderate throughput, potentially limiting industrial‐scale production. Turbulent jet mixing enables rapid, high‐volume production with reusable devices, offering greater scalability; however, operator skill and parameter optimization are critical to maintain batch consistency. Extrusion, while low‐cost and straightforward, lacks scalability and consistently lower protein retention limits its translational potential.

Considering in vivo applications, turbulent jet and microfluidic BNPs provide promising platforms for systemic administration, as they preserve membrane proteins necessary for targeted interactions and immune evasion. However, challenges remain, including controlling biodistribution, minimizing off‐target accumulation, avoiding rapid immune clearance, and ensuring reproducible pharmacokinetics. The ability to maintain structural stability and functional surface proteins under physiological conditions suggests that these BNPs could achieve improved circulation times and targeted tissue delivery, but preclinical studies will be required to validate their safety, biodistribution, and therapeutic efficacy.

In summary, this study provides a head‐to‐head comparison of BNP fabrication strategies, linking processing conditions to physicochemical stability, drug loading and release, protein incorporation, and functional performance. These insights offer mechanistic understanding and practical guidance for the rational design, scalable production, and translational development of next‐generation biomimetic nanocarriers.

## 4 Methods

### 4.1 SH‐SY5Y Culturing

The SH‐SY5Y cell line (CRL‐2266, ATCC) is a neuroblastoma cell line derived from a metastatic bone tumor. The cells were cultivated in a mixture of 50% DMEM and 50% Nutrient Mixture F12 HAM with Sodium B, supplemented with 10% heat‐inactivated FBS, 10 IU/mL penicillin, 0.1 mg/mL streptomycin, 1 µg/mL Amphotericin B, and 1% non‐essential amino acids. Fresh media was added every 3 days. Protein extraction utilized cells within passage numbers 29–32.

### 4.2 Membrane Protein Extraction and Quantification

Membrane proteins were extracted from SH‐SY5Y neuroblastoma cells (CRL‐2266, ATCC) using the “Triton X‐100‐based extraction protocol” [57]. Cells were washed with PBS and sequentially incubated with two extraction buffers: EB1 (10 mm PIPES, 100 mm NaCl, 5 mm EDTA, 300 mm sucrose, 0.015% digitonin) and EB2 (10% Triton X‐100), each supplemented with Halt Protease Inhibitor Cocktail (1:100, v/v). Incubations were performed at 4°C under gentle agitation, followed by centrifugation at 1000 × *g* (EB1) and 5000 × *g* (EB2). Supernatants containing solubilized membrane proteins were collected and stored at −80°C.

Protein concentration was determined using the Pierce BCA Protein Assay Kit (Thermo Fisher) according to the manufacturer's protocol, with absorbance measured at 562 nm (Infinite, TECAN).

### 4.3 NP Fabrication

NP were fabricated at a final lipid concentration of 10 mm. The lipid phase consisted of a mixture of DPPC, DOPC, and cholesterol at a molar ratio of 4:3:3, dissolved in ethanol. The aqueous phase contained either PBS (for liposomes) or an SH‐SY5Y protein extract diluted in PBS (for Neurosomes), with a protein‐to‐lipid ratio of 1:50 (w/w). For Dox encapsulating NPs, a 250 mm ammonium sulfate buffer at pH 4.4 was used instead of PBS. Before mixing, both the lipid and aqueous phases were pre‐heated to 45°C to ensure optimal lipid solubility and mixing conditions.

#### 4.3.1 Turbulent Jet Mixing

NPs fabricated using the turbulent jet mixing approach were fabricated using the NOVA IJM system (HELIX Biotech). The lipid and aqueous phases were simultaneously injected into the mixing chamber at a flow rate ratio (FRR) of 1:2 (lipid: aqueous) and a total flow rate (TFR) of 3 mL/min. This rapid mixing process enabled the spontaneous formation of NPs through controlled turbulent diffusion. To optimize mixing conditions, TFRs of 2.5 and 4 mL/min were also evaluated in Liposome samples; 3 mL/min was ultimately chosen based on the particle sizes obtained using the dynamic light scattering (DLS).

#### 4.3.2 Microfluidic Mixing

NPs fabricated using the microfluidic mixing approach were fabricated using the NanoAssemblr Ignite system (Cytiva). Lipid and aqueous phases were co‐injected into the microfluidic chip at a flow rate ratio of 1:2 and a total flow rate of 2.5 mL/min. The laminar flow conditions and controlled diffusion within the microchannels led to the spontaneous self‐assembly of liposomes with a narrow size distribution.

#### 4.3.3 Extrusion Following Ethanol Injection

For NPs fabricated via the extrusion following ethanol injection method, the lipid phase was rapidly injected into the aqueous phase (aqueous: lipid ratio of 9:1, v/v). This method initially produced a heterogeneous population of small unilamellar vesicles (SUVs) and multilamellar vesicles (MLVs). To obtain a uniform size distribution, the suspension was extruded sequentially through two 200 nm polycarbonate membranes (twice), followed by extrusion through one 200 nm membrane and two 80 nm membranes (twice), resulting in a homogeneous population of SUVs.

Following fabrication, all NP suspensions were dialyzed to remove ethanol and unencapsulated materials. For Neurosomes, dialysis was performed using a 1000 kDa MWCO membrane. For empty liposomes, a 12–14 kDa MWCO membrane was used. Dialysis was conducted in PBS at 4°C over three sequential cycles using a 1000‐fold excess of buffer for 1, 3, and 24 h, respectively.

For the in vitro experiments, rhodamine‐labeled NPs were used, incorporating 0.05% of 16:0 Liss Rhod PE (810158P, Sigma–Aldrich) into the lipid mixture.

### 4.4 Physiochemical Characterization

After the dialysis, the NPs were filtered through a 0.22 µm filter and were characterized for their size, polydispersity index (PDI), particle concentration, and zeta potential using dynamic light scattering (DLS) on a Malvern Zetasizer Ultra system. For size, PDI, and particle concentration measurements, samples were diluted 1:100 (v/v) in filtered PBS buffer. Each sample was measured in triplicate, with each measurement consisting of ten runs. Zeta potential was measured following a 1:100 (v/v) dilution of the samples in HPLC water. For each sample, three independent measurements were performed, and the final reported value represents the average of these three replicates.

### 4.5 Cryo‐TEM Imaging

Samples were prepared and imaged at the Center for Electron Microscopy of Soft Matter, Technion, Haifa. Before imaging, the NP samples were diluted 1:5 with HPLC‐grade water. Specimen preparation was performed using a Leica EM GP2 plunge freezer. The chamber was maintained at 25°C and 90% relative humidity to minimize evaporation. A carbon‐coated perforated polymer film supported on a 200‐mesh TEM grid was plasma‐treated using a PELCO EasiGlow glow discharger (Ted Pella Inc.) to increase surface hydrophilicity. In the plunge freezer chamber, each grid was held by tweezers, and a drop of the diluted sample was applied. Excess liquid was blotted to create a thin film, followed by vitrification through rapid plunging into liquid ethane maintained at −180°C. Vitrified grids were stored in liquid nitrogen until transferred under cryogenic conditions into the transmission electron microscope. Imaging was performed using a Thermo Fisher Scientific Talos 200 C high‐resolution TEM, equipped with a field emission gun (FEG) and operated at 200 kV. Samples were maintained at −180°C using a Gatan 626 cryo‐holder. A Volta phase plate was used to enhance image contrast.

### 4.6 NPs Stability in Physiological Conditions

NPs were mixed with SH‐SY5Y growth medium at a 1:4 ratio (NPs:medium) and incubated at 37°C for 6, 24, and 48 h. The 0 h time point corresponded to particles not subjected to medium incubation. After incubation, NPs were collected by ultracentrifugation at 100 000 × *g* for 1 h at 4°C. The supernatant was discarded, and the NP pellets were resuspended in PBS. The physicochemical properties of the NPs were then measured by DLS as described above.

### 4.7 Doxorubicin Active Loading and Encapsulation Efficiency Analysis

Doxorubicin Hydrochloride (Pharmaceutical Secondary Standard, Sigma–Aldrich) was added to the NPs at a final concentration of 1 mg/mL. The suspension was incubated for 60 min at 40°C under gentle agitation to facilitate remote loading. After incubation, unencapsulated doxorubicin was removed using overnight dialysis in PBS at 4°C. The encapsulation efficiency was determined using a fluorescence‐based doxorubicin quantification assay. NPs were lysed with 0.5% Triton X‐100, and the fluorescence of the released drug was measured using a microplate reader (excitation 495 nm, emission 590 nm). Concentrations were calculated using a standard curve of free doxorubicin and the encapsulation efficiency (%) was calculated as the ratio between encapsulated doxorubicin and the total amount added during loading.

### 4.8 Doxorubicin Release Assay

Doxorubicin release from liposomes and Neurosomes was assessed using a dialysis‐based diffusion assay. NP suspensions containing a known amount of encapsulated doxorubicin were loaded into dialysis bags (MWCO 10–12 kDa) and immersed in PBS (pH 7.4) at either 4°C or 37°C. Samples were collected from the dialysis bags at **8, 24,** 48, and 72 h to determine the remaining (non‐released) doxorubicin. Fluorescence was measured at 495 nm excitation and 590 nm emission, and concentrations were determined using a doxorubicin standard curve. The cumulative percentage of drug released was calculated relative to the initial encapsulated amount.

### 4.9 Proteomic Sample Preparation and Mass Spectrometry Analysis

Proteins incorporated into the NP were precipitated with 80% cold acetone overnight, washed three times with cold acetone, air‐dried, and resuspended in 8.5 m urea, 100 mm ammonium bicarbonate, and 10 mm DTT. Following protein quantification by Bradford assay, samples were reduced (60°C, 30 min), alkylated with 35.2 mm iodoacetamide (30 min, room temperature, dark), and digested with trypsin overnight at 37°C (1:50 enzyme‐to‐substrate w/w ratio), followed by a second 4‐h digestion (1:100 ratio). Peptides were desalted using HLB plates (Waters), dried, and resuspended in 0.1% formic acid. Samples were analyzed by LC‐MS/MS using a Q‐Exactive HFX mass spectrometer coupled to an Ultimate 3000 HPLC. Peptides were separated on a 30 cm C18 column using a 120‐min linear gradient (5%–28% acetonitrile) followed by a 15‐min ramp to 95%. MS data were acquired in positive mode (m/z 350–1200) with resolutions of 120 000 (MS1) and 20 000 (MS2), using HCD fragmentation of the top 20 ions.

Mass spectrometry data were analyzed using MaxQuant (version 2.4.2.0) [69]. Peptide identification was performed using the built‐in Andromeda search engine with specific digestion by trypsin, allowing up to two missed cleavages. Carbamidomethylation of cysteine was set as a fixed modification, and methionine oxidation and protein N‐terminal acetylation were set as variable modifications. The search was conducted against the *Homo sapiens* reference proteome downloaded from UniProt (August 2023, 20,424 entries) [70], using a target‐decoy strategy with a reversed decoy database. Peptide‐spectrum matches, peptides, and proteins were filtered to a false discovery rate (FDR) of 1% using a decoy‐based approach. A minimum peptide length of 7 amino acids was required, and the maximum precursor mass tolerance was set to default; quantification was based on razor and unique peptides. The “match between runs” feature was enabled to transfer identifications across samples with a match time window of 1 min. Reverse hits and potential contaminants were removed before further analysis. Statistical analysis of the MS results was carried out using the Perseus software platform version 2.0.10.0 [71] after log_2_ transformation. For differential expression analysis, only proteins with valid LFQ intensity values in all three replicates of at least one experimental group were included.

### 4.10 Proteomic Data Analysis

To evaluate membrane protein incorporation in BNPs, we classified the identified proteins based on their subcellular localization using Gene Ontology Cellular Component (GOCC) annotations. Proteins were considered membrane‐associated if they contained GOCC terms related to membrane localization, including “integral component of membrane”, “plasma membrane”, “cell surface”, and “membrane raft”. Proteins annotated with at least one of these terms were grouped as membrane associated. The relative abundance of membrane proteins in each sample was calculated by summing the total protein intensity of the membrane‐associated proteins (based on iBAQ values) and dividing it by the total protein intensity of all detected proteins (see the equation below). iBAQ is a label‐free proteomics metric that estimates the relative molar abundance of proteins by dividing the summed peptide intensities by the number of theoretically observable peptides for each protein, thereby allowing comparison across different proteins within a sample [62]. This analysis enabled a comparative evaluation of membrane protein incorporation efficiency across the different BNP fabrication methods.

$$\%\;Membrane\;Proteins=\left(\frac{Membrane\;Protein\;iBAQ}{Total\;iBAQ}\right)\times 100$$

Membrane protein incorporation into BNPs was evaluated by classifying proteins according to Gene Ontology Cellular Component (GOCC) annotations. Proteins annotated as “integral component of membrane”, “plasma membrane”, “cell surface”, or “membrane raft” were considered membrane‐associated. The relative abundance of membrane proteins was calculated by dividing the summed iBAQ intensities of membrane‐associated proteins by the total intensity of all detected proteins.

Heatmaps of representative membrane‐associated and non‐membrane proteins were generated using log2‐transformed, averaged LFQ intensities. Hierarchical clustering (Euclidean distance, complete linkage) was applied to group proteins and samples with similar patterns. This analysis allowed visualization of protein incorporation differences between BNP fabrication methods and provided mechanistic insights into targeted delivery to SH‐SY5Y cells.

### 4.11 SDS PAGE

The protein profile of the NPs was assessed by SDS‐PAGE. NP samples and the SH‐SY5Y protein extract were mixed with 5x sample buffer (MB01015, GenScript) and denatured at 95°C for 5 min to ensure NP disruption and release of associated membrane proteins. Samples were loaded with a molecular weight protein marker and separated by electrophoresis on an SDS‐polyacrylamide gel at 120 V for about 1 h. Following electrophoresis, the gel was washed and stained with Imperial Protein Stain (24615, Thermo Fisher) to visualize the protein bands.

### 4.12 Neurosomes Association Assay

The association of Neurosomes and control liposomes with SH‐SY5Y cells was evaluated using rhodamine‐labeled NP (810158P, Sigma–Aldrich). Cells (1 × 10⁴ cells/well) were seeded in 96‐well plates and incubated with NP at lipid concentrations of 0.25 and 0.1 mm for 4, 12, and 24 h at 37°C. Following incubation, cells were washed with PBS, and nuclei were stained with Hoechst 33342 (1:10 000 dilution; 62249, Thermo Fisher).

Fluorescence imaging was performed using a Cytation 5 microplate reader (BioTek) at 10x magnification. Image analysis was conducted using Gen5 software, where total rhodamine (RFP) fluorescence intensity in proximity to cell nuclei was quantified, along with the total nuclear area. To account for differences in intrinsic fluorescence between Neurosomes and liposomes, RFP intensity was normalized to the fluorescence of NP alone (measured under identical conditions). Final association values are reported as total normalized RFP intensity per total nuclear area.

### 4.13 MTT Viability Assay

SH‐SY5Y cells (1 × 10⁴ cells/well) were seeded in 96‐well plates and treated with NP (0.25 or 0.1 mm lipid) for 24 h at 37°C. After treatment, the media was replaced with 0.5 mg/mL MTT solution (M6494MTT, Rhenium) and incubated for 2 h. MTT was then removed, and DMSO was added to solubilize the formazan. Absorbance was measured at 570 nm, and cell viability was normalized to untreated controls.

### 4.14 Statistical Analysis

All experiments were performed with a minimum of three independent replicates unless otherwise stated. Data are presented as mean ± error of the mean (SEM). Statistical analyses were conducted using GraphPad Prism 10 (GraphPad Software, San Diego, CA, USA). For comparisons between two groups, an unpaired two‐tailed Student's t‐test was applied. For multiple group comparisons, one‐way or two‐way analysis of variance (ANOVA) was performed, followed by Tukey's or Dunnett's post hoc test, as appropriate. Differences were considered statistically significant at *p* < 0.05. Where applicable, correlation analyses were performed using Pearson's correlation coefficient to assess relationships between physicochemical parameters and functional outcomes. Graphical representations include bar charts with error bars denoting SEM as indicated in figure legends.

## Author Contributions

I.E., R.M., and A.Z. conceived the study and designed the experiments. I.E. fabricated the NPs using turbulent jet mixing and extrusion; R.M. fabricated the NPs using microfluidic mixing; and O.V. fabricated the NPs using extrusion. I.E. and R.M. performed nanoparticle characterization and stability assessments. R.M. designed, processed, and analyzed the proteomic experiments. I.E. performed and analyzed the in vitro experiments and SDS gel profiling. I.E., R.M., and A.Z. analyzed the data and co‐wrote the manuscript. A.Z. provided supervision, project administration, resources, and funding acquisition. All authors reviewed, revised, and approved the final version of the manuscript.

## Funding

Israel Science Foundation (164/22, 165/22); Norman Seiden Fellowship in Nanotechnology and Optoelectronics; Alon Fellowship; HORIZON EUROPE European Research Council MILKOSOMES No. 101115723; Russell Berrie Nanotechnology Institute, Technion‐Israel Institute of Technology NEVET 122317; Israel Cancer Association USA 20230006; Israel Cancer Research Fund 21‐206‐RCDA, 24‐111‐PG.

## Conflicts of Interest

The authors declare no conflicts of interest.

## Supporting information




**Supporting File**: smtd70440‐sup‐0001‐SuppMat.docx

## Data Availability

Research data are available in the supplementary information files. Additional raw data is available from the corresponding author on reasonable request.
