# Supplementary material for: Engineering Biomimetic Nanoparticle Performance Through Fabrication Method Selection: Turbulent Jet Mixing, Microfluidics, and Extrusion
Source: Small Methods. 2026 Jan 4;10(3):e01770. doi: 10.1002/smtd.202501770 (PMC12893306; doi:10.1002/smtd.202501770)
Supplement: Supplementary file 1 — Supporting File: smtd70440‐sup‐0001‐SuppMat.docx [file SMTD-10-e01770-s001.docx]

Supporting Information

**Engineering Biomimetic Nanoparticle Performance Through Fabrication Method Selection: Turbulent Jet Mixing, Microfluidics, and Extrusion**

Ilana Elizarov^†^, Rawan Mhajne^†^, Ofri Vizenblit, Assaf Zinger *


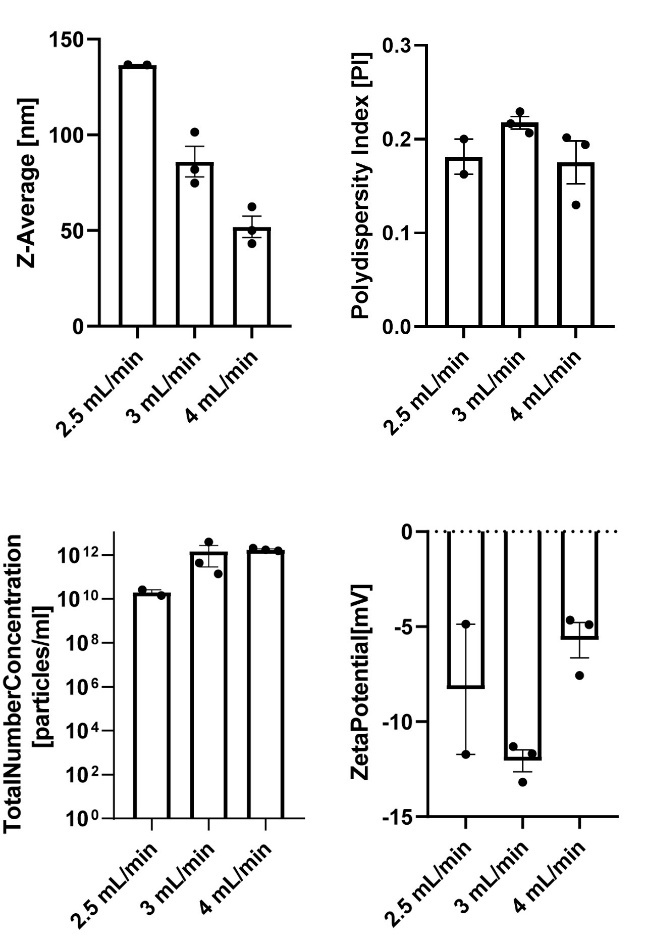


**Figure S1. Flow Rate Calibration for Liposome Fabrication Using the Turbulent Jet Mixing method.** Dynamic light scattering (DLS) characterization of liposomes produced at total flow rates of 2.5, 3, and 4 mL/min. Assessed physicochemical parameters included particle size, polydispersity index (PDI), particle concentration, and zeta potential. Data are presented as mean ± SEM (n = 3 for the 3 and 4 mL/min formulations; n = 2 for the 2.5 mL/min formulation).


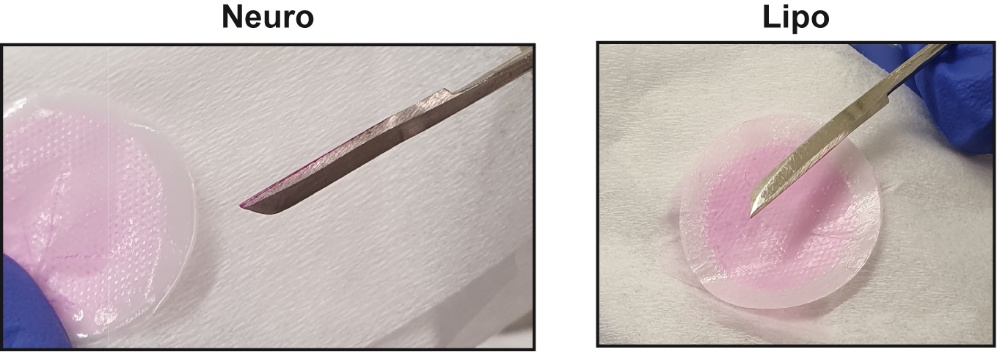


**Figure S2. Protein Deposition on Extruder Membrane.** Representative images of 80 nm polycarbonate membrane and spatula used following BNP extrusion, showing a visible pink smear in the Neurosome formulations. The color results from rhodamine-labeled lipids that were mixed with membrane proteins, indicating that protein shedding took place during the extrusion process.


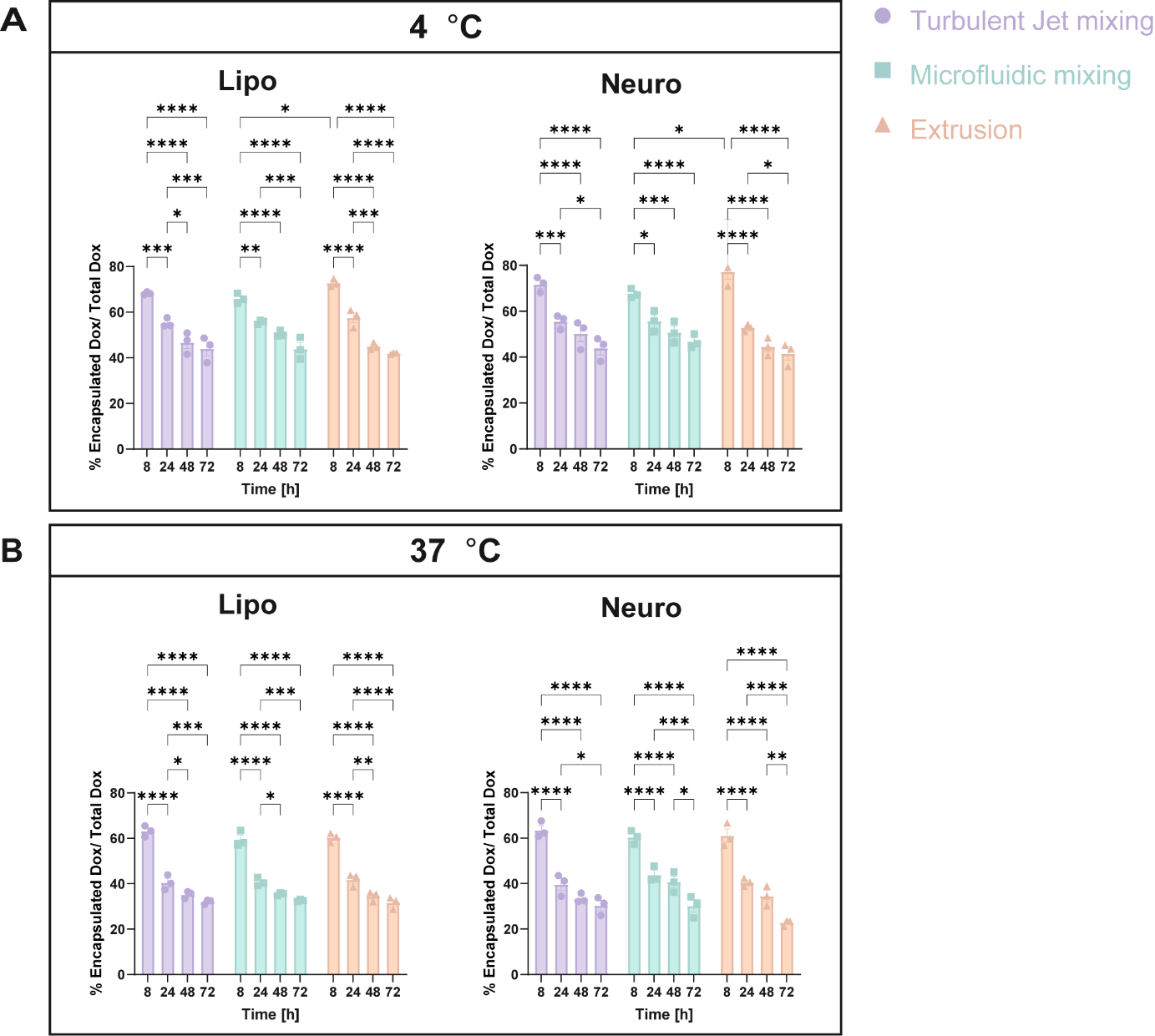


**Figure S3.** **Doxorubicin Release Profile of BNPs. (A)** Release profiles of Doxorubicin encapsulated BNPs measured at 4 °C **(A)** and 37 °C **(B)** over 8, 24, 48, and 72 h. Data are presented as mean ± SEM (n = 3 per formulation). Full statistical analysis was performed using two-way ANOVA followed by Tukey’s multiple comparisons test: *p < 0.05, **p < 0.01, ***p < 0.001, ****p < 0.0001.


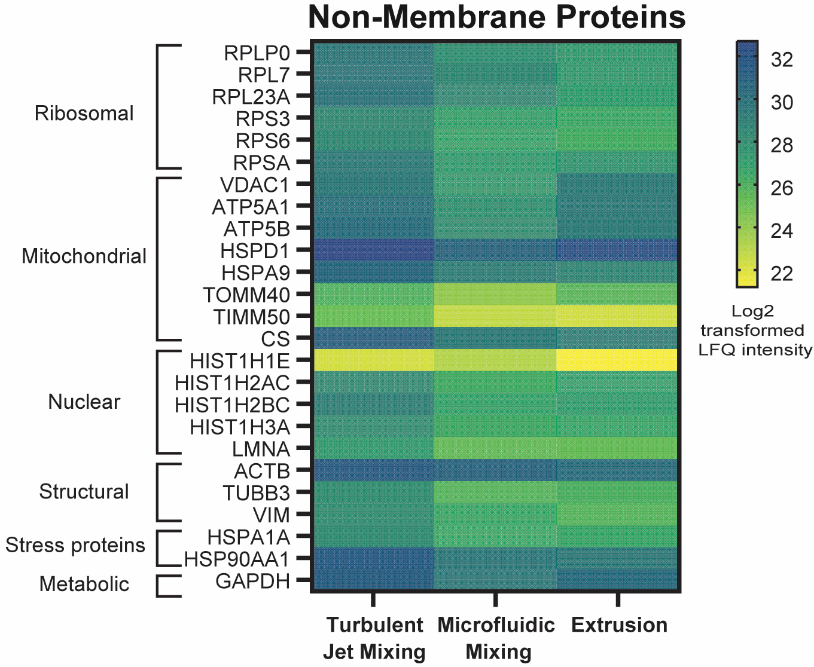


**Figure S4. Non membranal Protein Content of BNPs Fabricated by Different Methods.** Heatmap showing the relative abundance of representative non-membrane proteins incorporated into Neurosomes. Protein abundance is represented as the average of transformed LFQ intensity.


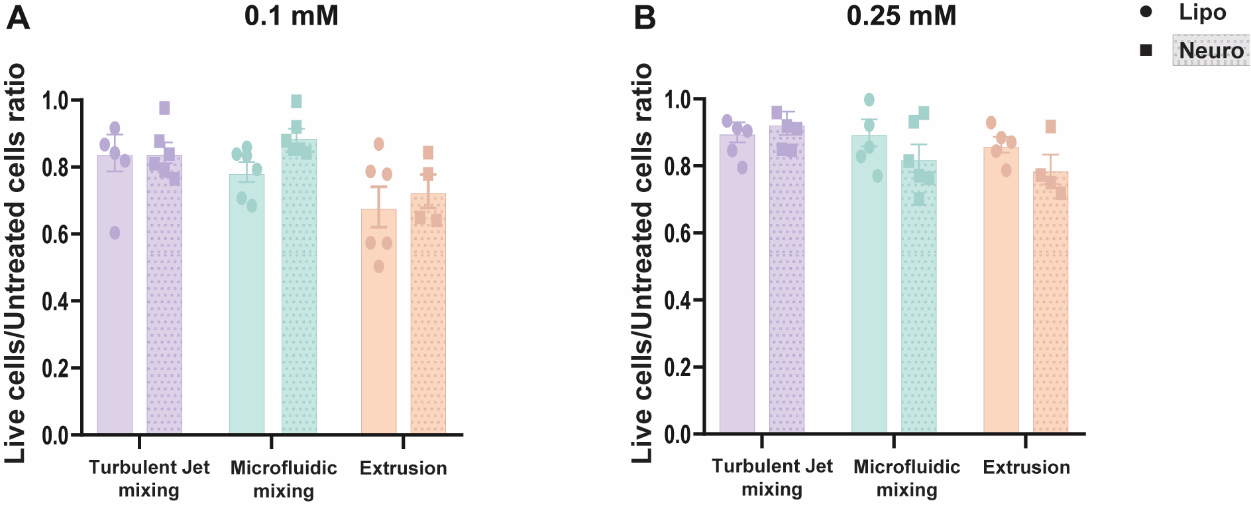


**Figure S5. BNP cytotoxicity Assay.** Neurosome cytotoxicity on SH-SY5Y cells was evaluated at 0.1 mM (**A**) and 0.25 mM (**B**) particle concentration following 24 h incubation using the MTT assay. Data is presented as mean ± SEM (n = 6 per formulation). Outlier values were excluded using the IQR method. Statistical significance was determined using two-way ANOVA followed by Tukey’s multiple comparison test.

**Table S1. Protein Content Values in BNPs**

| The Sample | Protein in the sample [ug] | Log 10 transformed data |
| --- | --- | --- |
| Turbulent Jet Mixing | 12.53 | 1.097951071 |
| Turbulent Jet Mixing | 14.51 | 1.161667412 |
| Turbulent Jet Mixing | 13.29 | 1.123524981 |
| Turbulent Jet Mixing | 14.95 | 1.174641193 |
| Turbulent Jet Mixing | 15.62 | 1.19368103 |
| Turbulent Jet Mixing | 15.11 | 1.179264464 |
| Microfluidic Mixing | 16.92 | 1.228400359 |
| Microfluidic Mixing | 16.34 | 1.213252052 |
| Microfluidic Mixing | 15.76 | 1.197556213 |
| Microfluidic Mixing | 17.48 | 1.242541428 |
| Extrusion | 1.8 | 0.255272505 |
| Extrusion | 4.917 | 0.691700208 |
| Extrusion | 4.068 | 0.609380944 |

**Table S2. Protein Incorporation Values in BNPs**

| Method | # of proteins | Percentage % | Proportion | arcsine square root transformation |
| --- | --- | --- | --- | --- |
| Turbulent Jet Mixing | 3639 | 97.25 | 0.97 | 1.404117861 |
| Turbulent Jet Mixing | 3655 | 97.68 | 0.98 | 1.417721023 |
| Turbulent Jet Mixing | 3668 | 98.02 | 0.98 | 1.429703199 |
| Microfluidic Mixing | 3651 | 97.57 | 0.98 | 1.414213204 |
| Microfluidic Mixing | 3662 | 97.86 | 0.98 | 1.424054866 |
| Microfluidic Mixing | 3599 | 96.18 | 0.96 | 1.374043126 |
| Extrusion | 3263 | 87.20 | 0.87 | 1.204907059 |
| Extrusion | 2777 | 74.21 | 0.74 | 1.038141342 |
| Extrusion | 3197 | 85.44 | 0.85 | 1.179233541 |

**Table S3. Membrane Protein Incorporation Values in BNPs**

| Method | Percentage 100% | Proportion | arcsine square root transformation |
| --- | --- | --- | --- |
| Turbulent Jet Mixing | 52 | 0.52 | 0.805403501 |
| Turbulent Jet Mixing | 53.1 | 0.531 | 0.816418058 |
| Turbulent Jet Mixing | 52.1 | 0.521 | 0.806404342 |
| Microfluidic Mixing | 53.1 | 0.531 | 0.816418058 |
| Microfluidic Mixing | 52.8 | 0.528 | 0.813412819 |
| Microfluidic Mixing | 53.7 | 0.537 | 0.822432016 |
| Extrusion | 50.8 | 0.508 | 0.793398505 |
| Extrusion | 51.5 | 0.515 | 0.800400414 |
| Extrusion | 50.2 | 0.502 | 0.787398169 |
